# Opioid prescription patterns in Germany and the global opioid epidemic: Systematic review of available evidence

**DOI:** 10.1371/journal.pone.0221153

**Published:** 2019-08-28

**Authors:** Bastian Rosner, Jessica Neicun, Justin Christopher Yang, Andres Roman-Urrestarazu

**Affiliations:** 1 Institute of Public Health, University of Cambridge, Forvie Site, Cambridge, England, United Kingdom; 2 Department of International Health, Care and Public Health Research Institute (CAPHRI), Faculty of Health, Medicine and Life Sciences, Maastricht University, Maastricht, The Netherlands; University of Mississippi Medical Center, UNITED STATES

## Abstract

**Introduction:**

Opioids are one of the most important and effective drug classes in pain medicine with a key role in most medical fields. The increase of opioid prescription over time has led to higher numbers of prescription opioid misuse, abuse and opioid-related deaths in most developed OECD (Organisation for Economic Co-operation and Development) countries around the world. Whilst reliable data on the prevalence of opioid treatment is accessible for many countries, data on Germany specifically is still scarce. Considering Germany being the largest country in the European Union, the lack of evidence-based strategies from long-term studies is crucial. The aim of this work is to review and summarise relevant published literature on the prevalence of opioid prescription in Germany to adequately inform health policy strategies.

**Methods:**

A systematic review of the epidemiology of opioid prescription in Germany was conducted, searching PubMed and Web of Science. Eligibility criteria were defined prior to conducting the search. Literature concerning Germany, published in English and German was included and the search was replicated by three independent researchers. Two levels of screening were employed. Disagreement was resolved by face-to-face discussion, leading to a consensus judgement.

**Results:**

Our electronic search yielded 735 articles. Reviewing titles and abstracts yielded 19 relevant articles. Three authors examined each article’s full text more closely and determined that twelve papers should be included. Of the twelve identified studies—with publication dates ranging from 1985 to 2016—six were retrospective cross-sectional studies and six were retrospective repeated-measures cross-sectional studies. Sample sizes ranged from 92,842 to ≈ 11,000,000 participants. Data sources of included studies showed vast heterogeneity. The reviewed literature suggested an increase in the number of patients with opioid prescriptions and defined daily doses of opioids per recipient in Germany over time. The majority of opioid prescriptions was used for patients with non-cancer pain. Opioid use was more common in older people, women and in the north of Germany. Fentanyl was shown to be the most prescribed strong opioid in outpatient settings in Germany, despite not being the first-line choice for chronic pain conditions. All data published before 2000—but none of the more recent studies—suggested an insufficient treatment of pain using opioids. There were no signs for a current opioid epidemic in Germany.

**Conclusions:**

Despite some limitations of the review and the heterogeneity of studies, it can be stated that the number of opioid prescriptions overall as well as the number of people receiving opioid treatment have increased over time. Most prescriptions were found to be for strong opioids and patients with non-cancer pain. Even though patterns of opioid prescription follow trends observed in other developed countries, there are no signs of an opioid epidemic in Germany. Therefore, this review could currently not find a need for urgent health policy interventions regarding opioid prescription practices. However, critical gaps in the literature remain and more research is needed to make more reliable judgements.

## Introduction

Opioids are one of the most important and effective drug classes in pain treatment [1–4], with a key role in modern anaesthesia, palliative care, emergency medicine and specialised pain management [3, 5, 6]. In 2016, North America, Oceania and Western Europe reported an average consumption of over 10,000 defined daily doses (DDD) of opioid analgesics [7]. Opioids pose a serious risk of addiction and abuse. Their long-term use still remains one of the biggest concerns about opioid treatment, since higher doses and prolonged continuous use increase the probability of adverse effects, habituation and dependence [8–12]. What was seen by some as the unmet need for pain management in chronic pain patients, caused by reluctance to use opioids considering their addiction potential [11, 13–15], has recently become an upsurge in numbers of opioid prescriptions through pharmaceutical market access strategies and policy-making. This is affecting G20 (Group of 20) countries severely and especially so in chronic non-cancer pain (CNCP) patients [12, 16–18]. The increase in opioid prescription over the past decade has led to higher numbers of prescription opioid misuse, abuse and opioid-related death cases in most developed OECD (Organisation for Economic Co-operation and Development) countries around the world [16, 19]. For example, the Global Burden of Disease Study showed a 22.3% increase in global opioid use disorder-related DALYs (disability-adjusted life years) between 2005 and 2015 [20]. Drug use disorders ranked as the 8^th^ most common cause of premature death in the US in 2016 [21]. The Canadian government reported an increase of 81% in accidental deaths involving fentanyl or fentanyl analogues between 2016 and 2017 [22]. Hence, there has been a shift from under-treatment of pain observed in the second half of the 20^th^ century, to an opioid crisis linked to over-prescription as part of pain management strategies [23]. In the US, roughly 21–29% of patients with opioid prescriptions for chronic pain misuse them and an estimated 4–6% of misusers eventually shift to heroin use [24–26]. In 2013, it was reported that about 2.4% of Australians aged 14 or older had used opioids for non-medical reasons at least once in their lives [27].

Whilst reliable data on the prevalence of opioid treatment is accessible for many countries, data on Germany is specifically sparse, despite it being the largest national economy in Europe, the fourth-largest by nominal GDP in the world, and fifth-largest by GDP (PPP) [28]. In 2017, Germany accounted for 28% of the euro area economy according to the International Monetary Fund, while having the largest population in the European Union (82 million people) [21, 28]. Consequently, and following the US and other developed economies, the opioid epidemic has recently become an issue of public debate, with concerns Germany might be following trends of other developed countries towards an opioid crisis [29, 30]. According to the UN’s 2017 report on narcotic drug use, Germany has the second highest opioid consumption of the 20 most populous countries in the world (28,842 DDD/1 million people/day; based on sales data) [7].

Pain medication prescription in Germany is based on national guidelines published by the AWMF (Arbeitsgemeinschaft der Wissenschaftlichen Medizinischen Fachgesellschaften e.V.). A guideline for the long-term use of opioids in the treatment (LTOT) of non-tumour pain was published in 2009 [31]. However, there is a multitude of guidelines concerning opioid prescription in different healthcare settings (e.g. palliative care, chronic-cancer pain, pain management for children) in Germany. As a result, prescribing opioids according to the appropriate guideline in place remains difficult. Thus, it is crucial to understand the current prevalence of opioid prescription in Germany to avoid a further rise of opioid misuse and opioid-related disorders. So far, the only effective approach to this national (and international) public health problem has been to design primary population level data collection strategies to analyse current and past trends in order to develop sufficient long-term prevention strategies.

### Aim

Considering the lack of evidence-based strategies from long-term studies on the German population in relation to the administration of opioids [14], the aim of this paper is to present results of a systematic review of literature concerning opioid prescriptions among outpatients in Germany. This study will assess the extent of opioid use for different health conditions, describe populations treated with prescription opioids, and characterise prescription patterns over time. Furthermore, it will use gathered information to provide recommendations for health policy. The review will focus on prescription of all types of opioids.

### Definition and classification of opioids

According to the World Federation of Societies of Anaesthesiologists, an opioid is “any naturally occurring, semi-synthetic or synthetic compound that binds specifically to opioid receptors and shares the properties of one or more of the naturally occurring endogenous opioids.” Opiates are defined as “any naturally occurring opioid derived from opium (e.g. morphine)” [32]. Opioids may be classified according to: (i) their analgesic potency, (ii) their origin and (iii) their action at the opioid receptor (Table 1) [32, 33].

**Table 1:** Classification of opioids used in Germany. Adapted from Trivedi et al. [32].

Potency	Origin	Function
**Strong** • Morphine (17.9%)* • Pethidine • Fentanyl (32.3%) • Alfentanil • Remifentanil • Sufentanil • Oxycodone (26.8%) • Piritramide • Hydromorphone (10.5%) • Tapentadol (2.9%) **Intermediate** • Buprenorphine (8.2%) • Nalbuphine • Tillidine Weak** • Codeine • Tramadol	**Naturally occurring** • Morphine • Codeine • Papavarine • Thebaine **Semisynthetic** • Diamorphine (Heroin) • Dihydrocodeine • Buprenorphine • Oxycodone • Hydromorphone **Synthetic** • Pethidine • Fentanyl • Alfentanil • Sufentanil • Methadone • Levorphanol • Piritramide • Tapentadol • Tillidine	**Pure agonists** • Morphine • Fentanyl • Alfentanil • Remifentanil • Sufentanil • Oxycodone • Piritramide • Hydromorphone • Tapentadol • Tillidine **Partial agonist** • Buprenorphine **Agonists-antagonists** • Nalbuphine **Pure Antagonists** • Naloxone • Naltrexone

* Numbers in brackets present the market share of the respective opioid according to packages sold for patients of statutory health insurances in Germany in 2011 (1.4% of market share devoted to “other opioids”). (Kieble M., 2012)

** Codeine and Tramadol do not require a special opioid prescription but can be obtained with a standard prescription.

### Opioid prescription laws and German guidelines for opioid therapy

Opioid prescription in Germany is tightly regulated by the Narcotic Drugs Prescription Ordinance (Betäubungsmittelverschreibungsverordnung, BtMVV) and by the German Narcotic Drugs Act (Betäubungsmittelgesetz, BtMG) which entered into force in 1992 [34].

The BtMVV gives detailed information on prescription rules (maximum quantities of opioids prescribed within a timeframe, maximum amount of different opioids prescribed at once) [35]. In Germany, all opioids must be prescribed by a medical doctor, a veterinarian or a dentist [34]. All opioids—except Tramadol and Codeine (normal prescription only)—require special narcotic prescriptions known as “BtM”-prescriptions [36].

Most opioid therapy regimens in Germany are based on the WHO guidelines for cancer pain treatments published in 1986 and are therefore following the WHO analgesic ladder [37]. This applies for all cancer pain treatments as well as for treatment of acute pain conditions.

In 2008, a guideline for long-term treatment of CNCP was published. The guideline established general indications and contraindications for opioid analgesic treatment for four weeks or longer. It also comments on how the treatment should be conducted, basing these recommendations on detailed analyses of the evidence and structured consensus formation [38].

## Methods

### Eligibility criteria

An important aspect when talking about drug therapy is the duration of treatment, since medical professionals distinguish between chronic or long-term (> 3–6 months) and acute or short-term (< 3–6 months) treatment [39]. Both, studies on chronic and acute pain management were included in this review. Also, studies on pain management in cancer and non-cancer patients were reviewed respectively.

Although most adult hospital patients receive are given opioids at least once during their hospital stay, no formal prescriptions are used. Instead, opioid use is recorded in so called opioid books, which makes assessment of opioid users in inpatient settings difficult [35]. Hence, this study focuses on outpatient settings only.

The following inclusion and exclusion criteria were established prior to the literature search:

- Medical setting: Only studies analysing data on prescription of opioids in outpatient settings were included.
- Participants: Only studies analysing data on use of prescription opioids among adults, regardless the underlying cause of the initial treatment, were included.
- Class of opioids: Studies analysing data on general opioid prescription or prescription of a certain class of opioids were included.

Two additional *a priori* exclusion criteria were established, excluding studies focused on children or adolescents, and those strictly referring to one specific opioid only (e.g. tramadol, fentanyl). Furthermore, language as a study reporting attribute was also defined as an inclusion/exclusion criterion (Table 2).

**Table 2:** Inclusion and exclusion criteria for study selection.

Inclusion criteria	Exclusion criteria
1. Full-text accessible at University of Cambridge 2. Language: English & German 3. Geographic area: Germany 4. Epidemiological data stating prevalence and/or incidence of outpatient opioid prescription 5. Studies on general Opioid prescription/ prescription of certain groups of opioids	1. Full-text not accessible at University of Cambridge 2. Languages other than English or German 3. Studies conducted in other German-speaking countries 4. Studies focused on children or adolescents 5. Studies strictly referring to one specific opioid (e.g. tramadol, fentanyl) 6. Studies solely referring to hospital opioid use 7. Studies (reviews) exclusively reporting results from papers already included in the review

### Literature search strategy

**Fig 1:**
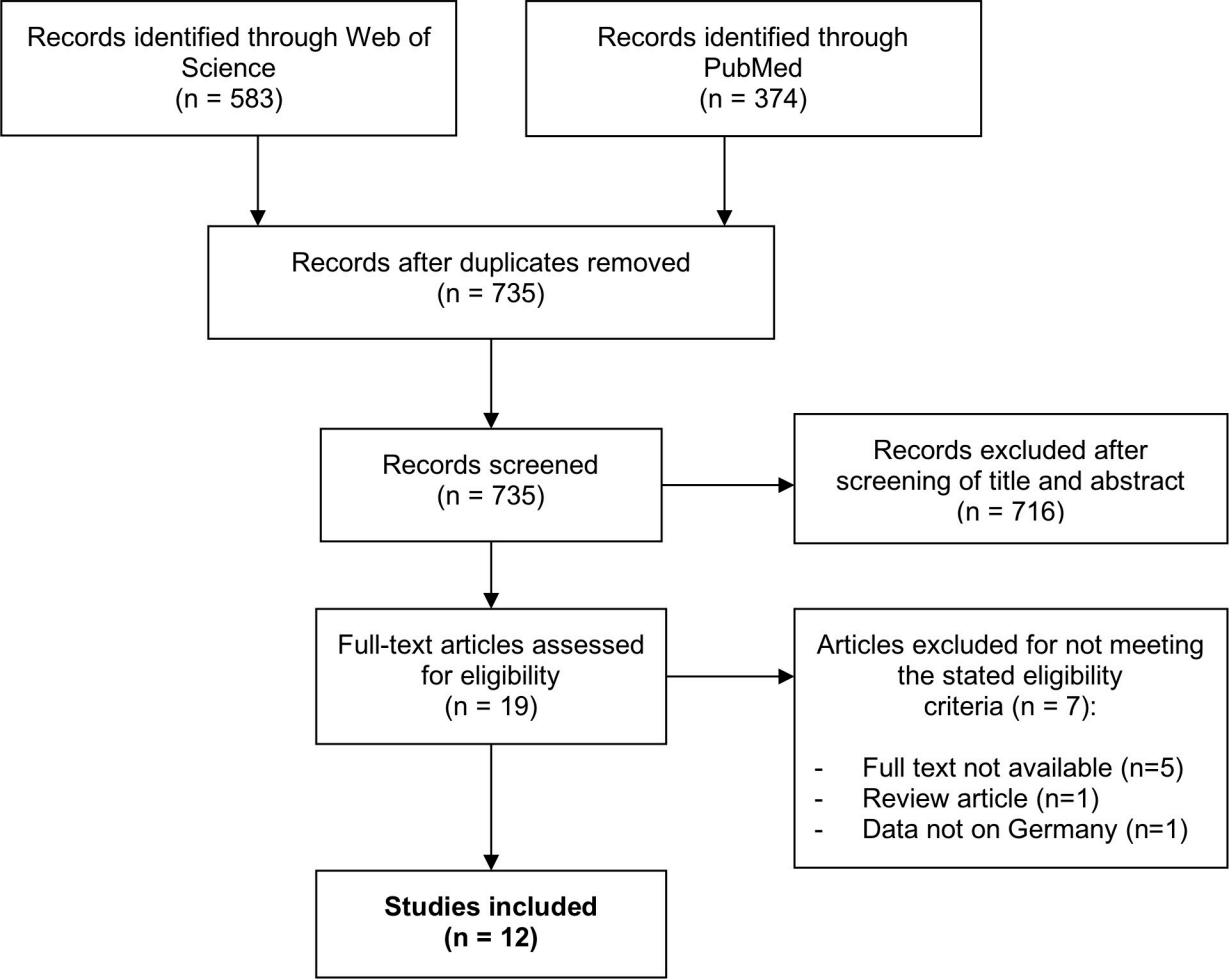
Literature search strategy.

A systematic literature search was carried out on 21 November 2018 using PubMed and Web of Science as primary data sources (Table 3 and Fig 1). Studies were selected upon meeting predefined eligibility criteria (Table 2). Additionally, a web search engine (Google.com) was employed to also include grey literature. Two levels of screening by three independent researchers (B. Rosner, J. Neicun, J. Yang) were used on all citations. Our electronic search yielded 735 articles. We reviewed titles and abstracts and excluded all articles that clearly did not meet our inclusion criteria. This process yielded 19 articles, which were retrieved for more thorough investigation in a second step. In-depth examination of the articles’ titles, abstracts and—if needed—full texts was conducted by all three investigators, resulting in a final selection of 12 articles. At all stages of the selection process, disagreements between reviewers were resolved by face-to-face discussion, eventually leading to a consensus judgement.

**Table 3:** Search terms used in database search.

Database	Search Strategy
**PubMed**	1. prescription [MeSH] **OR** prescription* **OR** prescrib* AND 2. analgesics, opioid [MeSH] **OR** alkaloids, opiate [MeSH] **OR** opioid* **OR** opiate* AND 3. germany [MeSH] **OR** german* No filters used.
**Web of Science**	1. prescription* **OR** prescrib* AND 2. Opioid* **OR** opiate* **OR** sedative* **OR** analgesic* AND 3. german* No filters used.

Due to the small number of included studies and the heterogeneity in study methodology, the appropriateness of a meta-analysis had to be critically questioned and therefore, was not included as part of this study.

### Quality assessment

The quality of studies was assessed using the EPHPP (Effective Public Health Practice Project) quality assessment tool for quantitative studies (S1 Table). The tool generates a total quality score between one (strong) and three (weak) based on six sub-scores, assessing components of internal and external validity.

Only three of the studies were awarded one point and therefore a strong rating. One study was given moderate rating and eight were rated weak. This was mainly due to assumed selection bias inherent to the nature of the data source (i.e. statutory health insurance that does not account for privately insured patients, private prescriptions and patients of other insurances, patients changing health insurance). Thus, no credible inferences about the entire German population may be drawn from these papers. Likewise, no validity and reliability assessment for data collection tools could be performed. As a result, four of the included cross-sectional studies were awarded moderate component ratings for study design, and eight were rated weak.

Overall, the evidence base was rated as low. However, the applied tool might not be ideal for rating the quality of studies on secondary data such as insurance claims data and medical records since some of the assessed components seem to be inappropriate (e.g. study design, data collection methods or blinding).

## Results

Twelve studies were found eligible for inclusion in this review (Table 4) with the search for grey literature not revealing any additional relevant data. The agreement percentage between raters was substantial at 83.3% (Cohen’s kappa = 0.79). Publication dates ranged from 1985 to 2016, six were retrospective cross-sectional studies [8, 40–44] and six were retrospective repeated-measures cross-sectional studies [13–15, 45–47]. The six studies including national data on Germany did not analyse data from the same time period. Thus, no data overlap happened. Among the five studies analysing data from overlapping periods, two used data from different sources [14, 15], one did not give details of its data source [41] and two may have presented some temporal/geographical overlapping [13, 45]. However, this overlap was considered to broaden the evidence base while bringing validity to the results, so both studies were included in the review respectively.

**Table 4:** Summary of studies included in the systematic review.

#	Reference	Journal	Year	Region	Age Range (years)	Total # of patients	Study period	Type of data / Primary data source	Study type	Descriptive measures reported in studies
**1**	**Buth et al. [15]**	Bundesgesundheitsblatt-Gesundheitsforschung-Gesundheitsschutz	2017	Schleswig-Holstein, Hamburg, Bremen, Nieder-sachsen	Not given	≈ 11,000,000	2005 to 2011	Prescription data / North German Pharmacy Computing Centre (Norddeutsches Apo- thekenrechenzentrum, NARZ)	Retrospective repeated measures cross-sectional study	Prevalence of patients with opioid prescriptions (subgroups analysis of age groups, mean duration of treatment/age groups), prevalence of LTOT, mean DDD/patient
**2**	**Hoer et al. [41]**	Schmerz	2008	Germany	Not given	1,534,034	2000 to 2003	Insurance claims data / Statutory health insurance in Germany	Retrospective cross-sectional study	Prevalence of patients with opioid prescriptions
**3**	**Hoffmann et al. [40]**	Schmerz	2012	Germany	Not given	9,100,000	2011	Insurance claims data / BARMER GEK	Retrospective cross-sectional study	Strong opioids only: Prevalence of patients with opioid prescriptions, DDD/user (subgroup analysis for different opioids)
**4**	**Ihle et al. [45]**	Pharmacoepidemiology and Drug Safety	2012	Hesse	Not given, mean age 43.9 (2000) 46.4 (2009)	326,598 (2000) 264,982 (2009)	2000 to 2009	Insurance claims data / AOK Hesse	Retrospective repeated measures cross-sectional study	Prevalence of patients with opioid prescriptions, DDD/user, DDD increase
**5**	**Jacob et al. [43]**	Postgraduate Medicine	2018	Germany (France) (UK)	≥ 18	4,270,142	2016	Data from patient records / Disease Analyzer database (QuintilesIMS)	Retrospective cross-sectional study	Prevalence of patients with pain medicine prescriptions (opioids as subgroup)
**6**	**Lindena et al. [46]**	Schmerz	1996	Germany	Not given	1,218,436	1990 to 1996	Market share data, prescription data, survey data, data from a questionnaire / Der Deutsche Pharmamarkt (DPM), Mediplus, telephone survey, questionnaire for clinicians	Retrospective repeated measures cross-sectional study	Strong opioids only: Prevalence of patients with opioid prescriptions
**7**	**Marschall et al. [8]**	European Journal of Pain	2016	Germany	Any age	870,000	2012	Insurance claims data / BARMER GEK	Retrospective cross-sectional study	LTOT for CNCP only: Prevalence of prescriptions for CNCP among all insureds, Prevalence of insureds with high-dose opioids among LTOT
**8**	**Schubert et al. [13]**	Deutsches Ärzteblatt International	2013	Hesse	Not given, mean age 43.9 (2000) 46.3 (2010)	326,554 (2000) 265,213 (2010)	2000 to 2010	Insurance claims data / AOK Hesse	Retrospective repeated measures cross-sectional study	Prevalence of patients with opioid prescriptions, DDD/user, DDD increase
**9**	**Sorge et al. [47]**	Schmerz	1990	Hannover	Not given	322,467 (1985) 325,506 (1988)	1985 & 1988	Insurance claims data / AOK Hannover	Retrospective repeated measures cross-sectional study	Strong opioids only: Prevalence of patients with opioid prescriptions, total DDD Germany
**10**	**Werber et al. [14]**	Pain Physician	2015	Germany	Not given, mean age 42.2	6,800,000	2006 to 2010	Insurance claims data / BARMER GEK	Retrospective repeated measures cross-sectional study	Prevalence of patients with opioid prescriptions, total DDD CNCP & CCP
**11**	**Willweber-Strumpf et al. [44]**	Schmerz	1992	Bochum	Not given	92,842	1989 to 1990	Insurance claims data / AOK Bochum	Retrospective cross-sectional study	Strong opioids only: Prevalence of patients with opioid prescriptions
**12**	**Zenz et al. [42]**	Journal of Pain and Symptom Management	1995	West Germany	Not given	1,104,435	1990 to 1993	Computerised patient records data / 330 practices in West Germany	Retrospective cross-sectional study	Strong opioids for cancer pain only: Prevalence of patients with opioid prescriptions

Sample sizes differed between 92,842 and ≈ 11,000,000. All reviewed studies used secondary data as their main data source: eight studies used health insurance claims data [8, 13, 14, 40, 41, 44, 45, 47], one used prescription data from a pharmacy computing centre [15], one from a disease analyser database [43], one from computerised patient records [42], and one from mixed sources (clinical and market research) [46]. Among the eight publications using health insurance claims records as data source, three obtained their data from BARMER GEK [8, 14, 40], four from regional branches of the AOK [13, 44, 45, 47], and one did not state the name of the statutory health insurance [41].

Six studies investigated the prevalence of opioid use regardless of treatment or opioid class [13–15, 41, 43, 45]. Four studies specifically researched the prevalence of treatment with strong opioids [40, 44, 46, 47]. Marschall et al. [8] focused on the prevalence of opioid use among long-term treatment for chronic non-cancer pain patients, while Zenz et al. [42] solely investigated patients with malignant diagnoses (cancer).

### Prevalence

**Fig 2:**
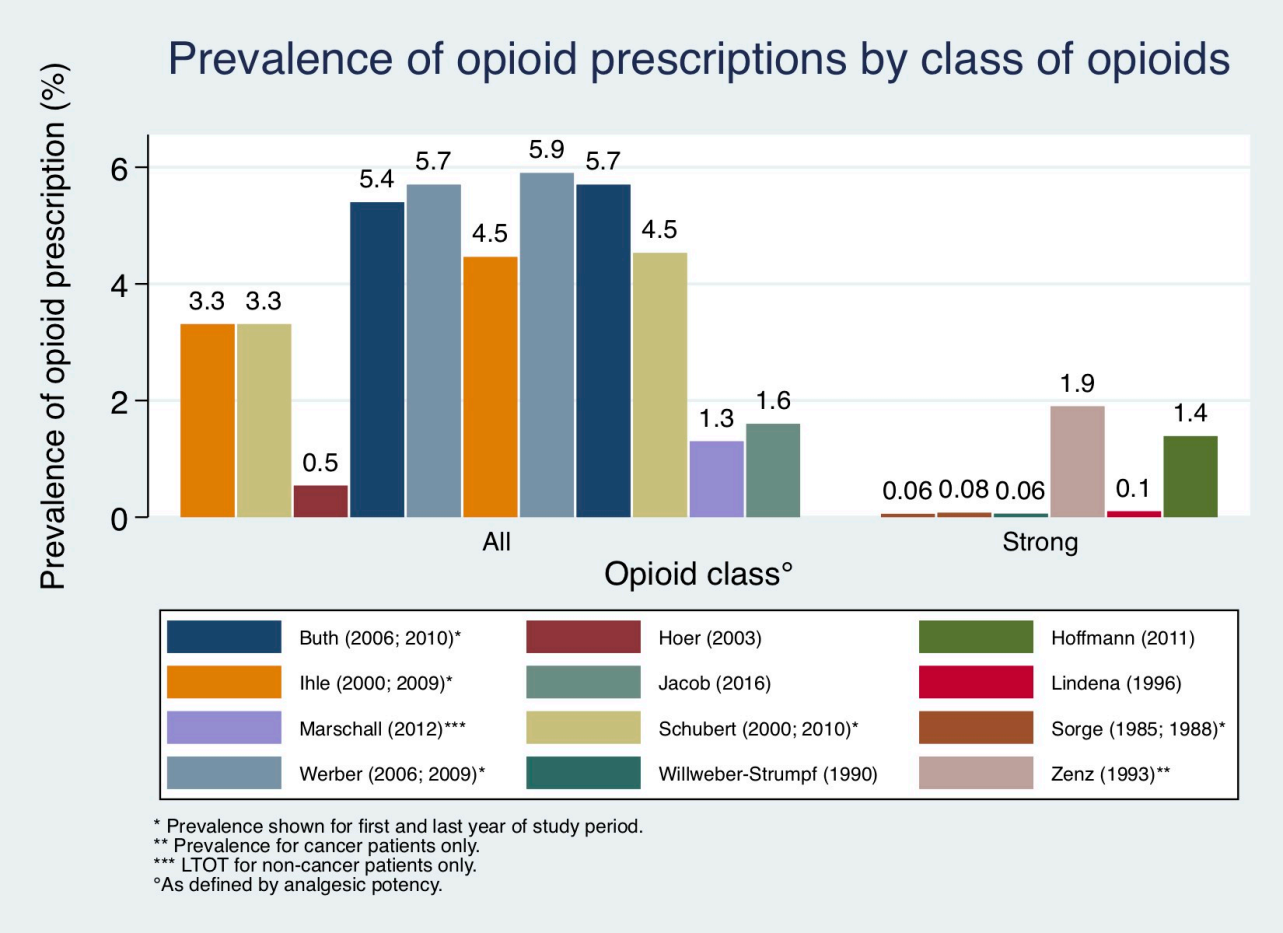
Prevalence of opioid prescription by opioid class included in study.

All studies used period prevalence as their primary outcome measure, defined as the proportion of a population using a drug within a certain time period (Fig 2 and Table 5) [48]. Six studies reported the prevalence for patients with any opioid prescriptions within their samples ranging from 0.54% to 5.7% [13–15, 41, 43, 45]. One study calculated the prevalence of LTOT prescriptions for CNCP among all insureds at 1.3% [8]. Four papers reported the prevalence for patients with prescriptions for strong opioids between 0.057% and 1.39% [40, 44, 46, 47]. Zenz et al. [42] calculated in 1995 that 1.9% of cancer patients received strong opioids.

**Table 5:** Comparison of descriptive measures.

#	Reference	Patients with opioid prescription (%)	Mean duration of treatment (days)	Prevalence of LTOT among patients with opioid prescription (%)	DDD/user	Mean DD of LTOT (mg)	Treatment for CNCP/CCP (%)	Additional measures
**1**	**Buth et al. [15]**	**5.4** (2006)– **5.7** (2010)	**107** (2006)– **114** (2010)	**19.2** (2006)– **21.2** (2010)	**1.0** (2006)– **1.0** (2010)	-	-	-
**2**	**Hoer et al. [41]**	**0.54**	-	-	-	-	-	-
**3**	**Hoffmann et al.[40]** *	**1.39**	-	-	2.086***	-	-	Opioid prescribed most: Fentanyl, **40.8% of DDD**
**4**	**Ihle et al. [45]**	**3.31** (2000)– **4.46** (2009)	-	-	2000–2009: **+67%**	-	**82.7/17.3** (2000) **79.4/20.6** (2009)	DDD increased by **+122.6%**
**5**	**Jacob et al. [43]**	**1.6**	-	-	-	-	-	-
**6**	**Lindena et al. [46]** *	**0.1**	-	-	-	-	**60.2/40.8**	**-**
**7**	**Marschall et al. [8]** ****	-	-	1.3**	-	**58**	-	Insureds with high-dose opioids among LTOT: **15.5%**
**8**	**Schubert et al. [13]**	**3.31** (2000)– **4.53** (2010) (+37%)	-	-	2000–2010: **+ 53.4%**	-	**80.6/19.4** (2000) **76.7/23.3** (2010)	DDD increased by **+109%**
**9**	**Sorge** **et al. [47]** *	**0.057** (1985) **0.075** (1988)	-	-	-	-	-	Total DDD Germany: **56 million** (1985)– **62 million** (1988)
**10**	**Werber et al. [14]**	**5.7** (2006)– **5.9** (2009) (+3.5%)	-	-	-	-	-	DDD CCP: **6,282,000** (2006), **8,087,000** (2009) DDD CNCP: **27,398,000** (2006), **32,391,000** (2009)
**11**	**Willweber-Strumpf** **et al. [44]** *	**0.059**	-	-	-	-	**17.76/82.24**	-
**12**	**Zenz et al. [42]**	**1.9** *****	-	-	-	-	**-**	-

* Strong opioids only

** Prevalence of LTOT prescriptions for CNCP among all insureds

*** DDD per insured, not per user

**** LTOT only

***** Strong opioids for cancer pain only

### Age and sex

Ten studies did not state an age group for participants included in their studies. Two studies reported a mean age between 43.9 and 46.3 years [13, 45]. Two papers provided sub-analyses for opioid prescription in specific age groups [8, 15]. Six of the included studies did not provide any gender-specific characteristics of prescriptions [15, 41, 42, 44, 46, 47]. Jacob et al. [43] did not analyse differences in prevalence of opioid prescription between males and females but did provide the prevalence of patients with pain medication prescriptions separately for males (28.9%) and females (30.3%). One study estimated that women are less likely than men to receive high-dose opioid prescriptions (adjusted OR 0.88; 95% CI: 0.77 to 0.99; p = 0.03) [8]. Werber et al. [14] solely described the peak age of opioid usage being different for men (40–45 years) and women (45–50 years). No further details were given to underpin this conclusion [14]. A sub-analysis for strong opioid use in 2011, provided by Hoffmann et al. [40], showed that 70.9% of new users of fentanyl patches were women.

Ihle et al. [45] reported higher prevalence of opioid prescriptions for women both in 2000 (males: 2.68%; females: 3.90%) and 2009 (males: 3.67%; females: 5.23%). Schubert et al. [13] showed higher prevalence of use of prescription opioids among women in all years observed. The latter also compared prevalence by sex for two years (2000 and 2010) for different stages of the WHO ladder. Again, prevalence was higher for women within all strata [13].

### Trends over time

Eight studies analysed prevalence of opioid prescription for a period longer than one year [13–15, 41, 42, 45–47]. Five of these studies analysed repeated measures and reported a slight increase in prevalence over time [13–15, 45, 47]. Despite reporting results for a study period of four years, three studies [41, 42, 46] only gave one overall estimate for opioid prescription prevalence (0.1%; 1.9%; 0.54% respectively). The biggest increase in prescription prevalence was reported by Schubert et al. (+37%) [13].

**Fig 3:**
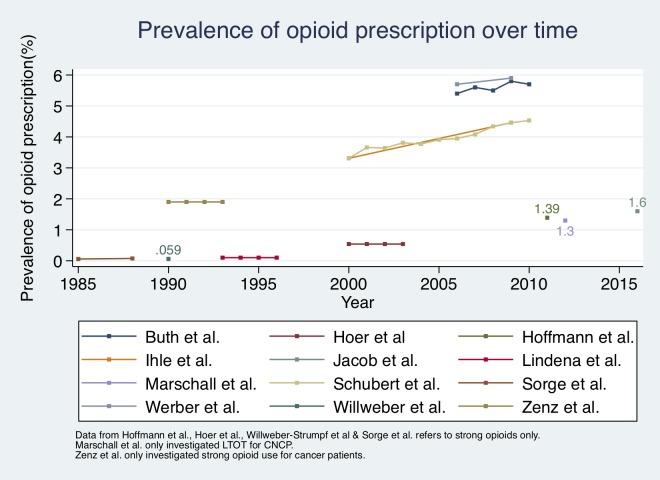
Comparison of opioid prescription prevalence over time.

Four studies calculated the prescription prevalence for one year only, ranging from 0.059% (strong opioids only) in 1990 to 1.6% in 2016 [8, 40, 43, 44] (Fig 3).

### Geographical differences

**Fig 4:**
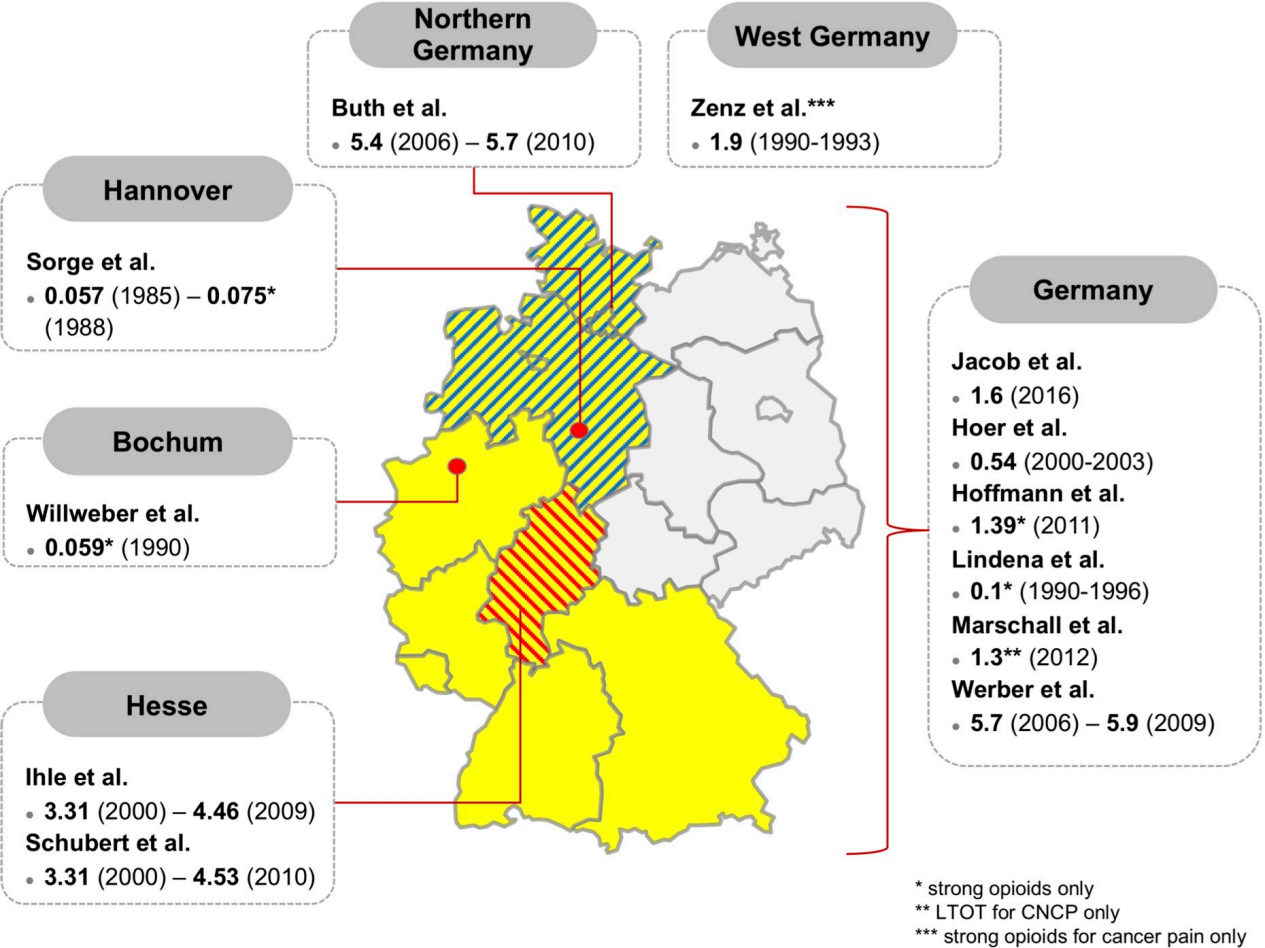
Geographical distribution of study population including prevalence of opioid prescription in % [49].

Fig 4 shows the geographical distribution of the included sample populations within Germany. Six studies referred to the entirety of the German population [8, 14, 40, 41, 43, 46], one study included data from West Germany only [42], two studies concerned the region of Hesse [13, 45] and one of Northern Germany [15]. Two studies were geographically focussed on the cities of Hannover [47] and Bochum [44] respectively.

**Fig 5:**
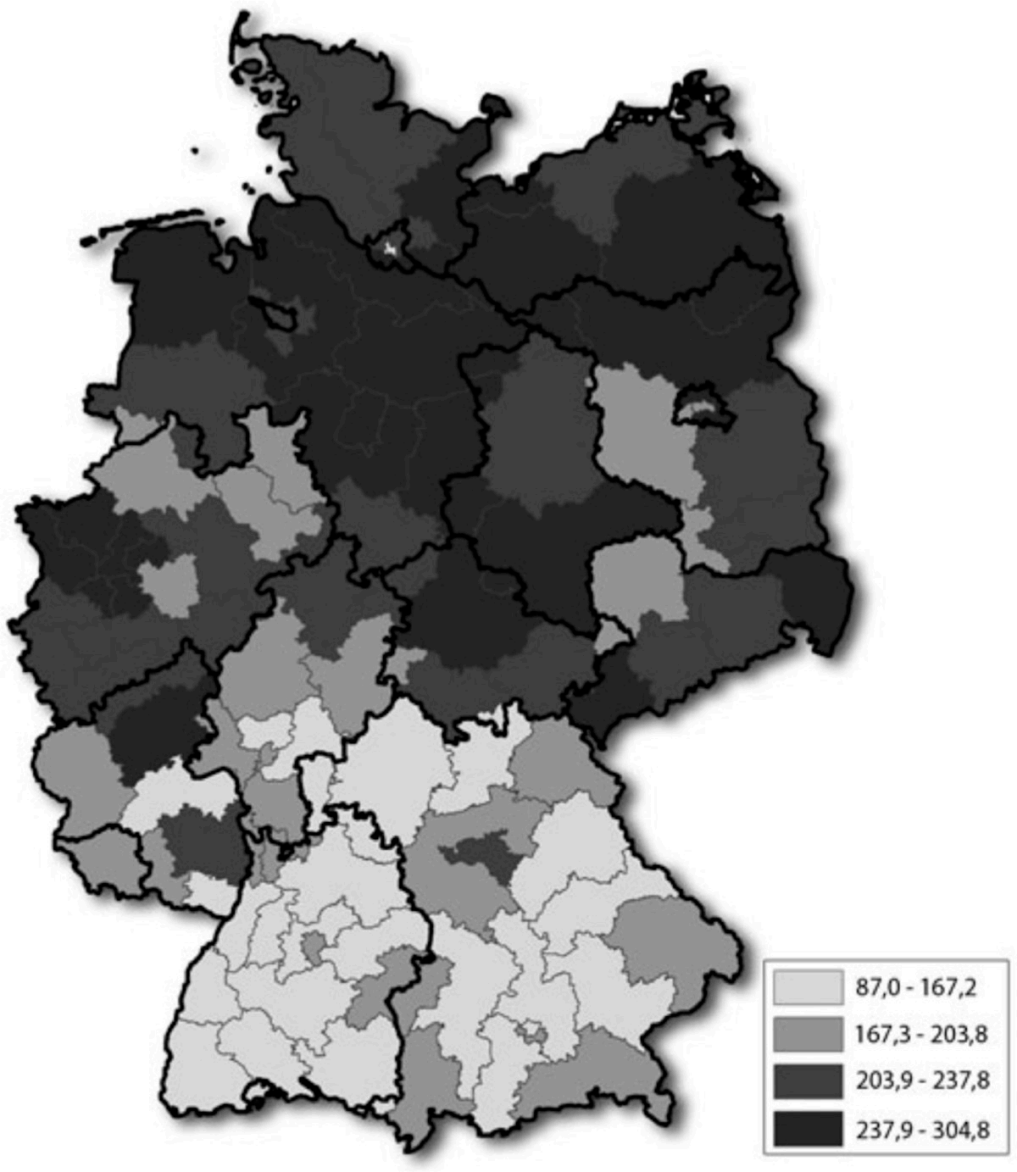
Prescription quantities of high-potency opioids in DDD/100 insureds according to postcode regions in 2011. (Hoffmann et al.) [40].

The only study reporting geographical patterns in (high-potency) opioid prescription within Germany was conducted in 2011 by Hoffmann et al. (Fig 5) [40]. Significant regional differences were found with regard to opioid prescription prevalence by state, ranging from 1.13% (Baden-Württemberg) to 1.67% (Lower Saxony) [40]. Similar differences were found with regard to opioid prescription quantities: smaller proportions were observed in the south (157.7 and 145.9 DDD/100 insureds for Bavaria and Baden-Württemberg respectively) compared to the north (259.5 DDD/100 insureds in Lower Saxony and 240.5 DDD/100 insureds in Mecklenburg-West Pomerania). Differences were even clearer when calculating numbers for postcodes rather than for states, with prescription volumes ranging from 87.0 DDD/100 insureds to 304.8 DDD/100 insureds.

### Diagnosis

**Fig 6:**
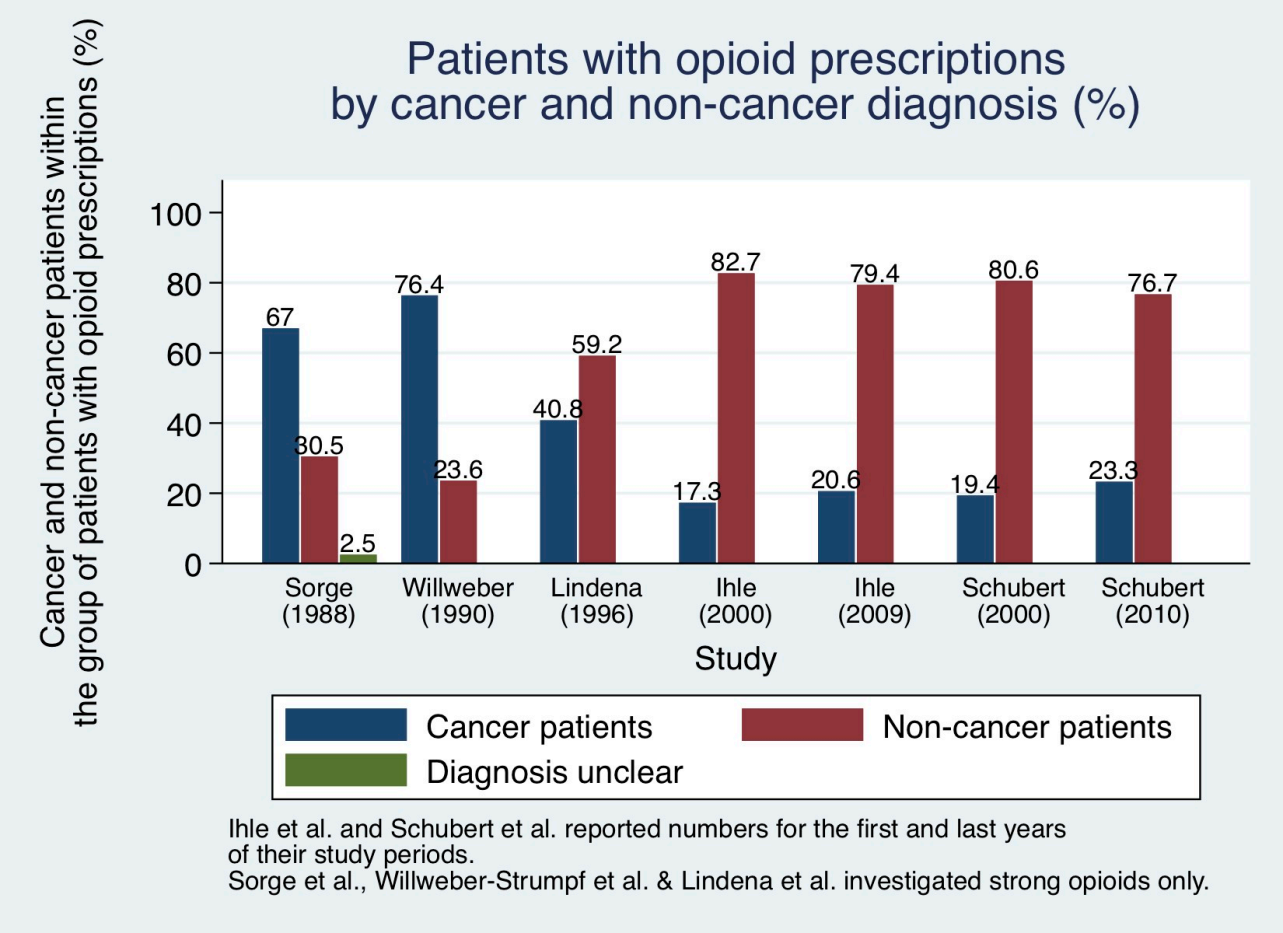
Proportion of cancer and non-cancer patients within the group of patients with opioid prescriptions. Confidence intervals not given.

A sub-analysis of underlying causes of opioid prescriptions was provided by seven of the studies [8, 13, 14, 44–47]. All seven studies distinguished between prescriptions for cancer and non-cancer pain. Five studies solely reported the fraction of all opioid prescriptions for every group [13, 44–47]. Sorge et al. and Willweber-Strumpf et al. [44, 47] reported significantly higher numbers of people with cancer diagnoses receiving opioid prescriptions in the late 80s, whereas Lindena et al., Ihle et al. and Schubert et al. [13, 45, 46] found that the majority of patients with opioid prescriptions did not have a malignant diagnosis (Fig 6).

Ihle et al. and Schubert et al. [13, 45] analysed opioids of different analgesic potency, and how treatment with each of them differed between cancer and non-cancer patients [13, 45]. Werber et al. [14] subdivided opioid treatment for cancer and non-cancer patients by strong and mild opioids [14]. They found that in 2006, 4.44% and 0.64% of all insureds received treatment with mild opioids for non-cancer and cancer conditions respectively. The numbers decreased for non-cancer patients to 4.21% and increased for tumour patients to 0.73% in 2009. Furthermore, they reported that—for mild opioids—most prescriptions in 2010 were issued for back pain (23.4%), spondylosis (9.3%) and gonarthrosis (8.5%). For strong opioids, most frequent diagnoses in 2010 were back pain (18.0%), unspecific pain (15.1%) and osteoporosis (9.3%). The prevalence of strong opioid prescriptions among insureds increased for both, non-cancer (2006: 0.75%, 2009: 1.01%, +34.7%) and cancer patients (2006: 0.33%, 2009: 0.42%, +27.3%) [14]. Marschall et al. [8] calculated prevalence of opioid use for non-cancer patients only. Despite including different medical conditions in their analysis as potential confounders, they only described the most frequent orthopaedic diagnoses associated with LTOT (low back pain, osteoarthritis). In addition, they also found nearly half of their sample to be diagnosed with somatoform pain disorder [8].

Zenz et al. [42] investigated the prevalence of strong opioid prescriptions for cancer patients and did not include numbers for non-cancer patients in findings.

## Discussion

The reviewed literature suggests an increase in the number of patients with opioid prescriptions and DDD of opioids per recipient in Germany over time [13–15, 45, 47]. Moreover, during the last decade, the majority of opioid prescription seems to have been used for patients with non-cancer pain, although the German guideline does not recommend opioids as a first-line therapy for CNCP [13, 31, 38, 45]. The use of prescription opioids tends to be more common in older people, women and in the north of Germany [8, 13–15, 40, 45]. Fentanyl seems to be the most prescribed strong opioid in outpatient settings in Germany despite not being the first-line choice for chronic pain conditions [14, 40, 41]. Globally, a pronounced trend towards strong opioids—particularly in non-cancer patients—is described [13, 14]. All data published before 2000 suggests insufficient pain management using opioids [42, 44, 46, 47]. Although studies’ findings show an increase in terms of patients with opioid prescriptions and DDD of opioids per recipient during the last decade, none of the more recent studies show signs for an opioid epidemic in Germany [8, 50]. In fact, despite a 30% increase between 2012–15, drug-related deaths in Germany (74% of which concern opioids) have remained relatively stable since 2006 overall [51].

### Strengths and limitations

This review has not been registered through PROSPERO prior to publication and thus, the risk of other reviews addressing the same question being published simultaneously cannot be ruled out. Only two databases were searched in order to find relevant studies for this review which reflects a potential selection bias. Searches were limited to titles and abstracts only. Relevant studies in which opioids were mentioned as a subgroup of pain medication may have been missed. Although no exclusion criteria were defined regarding time, no studies were found dating back to before 1990. This may be due to there being no register of older studies in the databases, but also to the political division of Germany that remained until 1989.

The unavailability of full-texts of possibly relevant studies may also be source of selection bias. The comparability of findings is limited by different case ascertainment strategies and the different regions within Germany studies are referring to. Also, this review is affected by publication bias since it exclusively relies on published papers. An overview of the strengths and limitations of data included in this review is presented in Table 6.

**Table 6:** Strengths and limitations of the included data.

Strengths	Limitations
• Studies in German and English were included in this review • Most studies used very large study samples (only four studies [13, 44, 45, 47] had samples of less than 500,000 patients) • All studies used the same opioid classification system (WHO) • All studies except one [8] used the prevalence of patients with opioid prescriptions as a primary outcome measure • The majority of the data derived from statutory health insurances which means that there is no recall or interviewer bias involved in the included retrospective studies	• Only three studies randomised their study sample to reduce confounding [8, 13, 45] • All of the included studies were retrospective • There is no national surveillance data on opioid consumption in Germany; studies rely solely on other data sources—mostly registries—which were not designed for study purposes in the first place and cannot represent the German population in its entirety (selection bias) • Eight studies [8, 13, 14, 40, 41, 44, 45, 47] had one statutory health insurance each (some of them the same) as primary data source to draw inferences about the German population which is a potential selection bias since it does not account for privately insured patients, private prescriptions & patients of other insurances, patients changing health insurances • Two studies [13, 45] got their data from exactly the same study sample and used the same methodology but reported results for different years • Only six studies referred to all of Germany [8, 14, 40, 41, 43, 46]; four studies referred to regions [13, 15, 42, 45] and two studies to cities [44, 47] within Germany to draw inferences about the German population (lack of generalisability) • Different opioid classes and sometimes even different opioids within one opioid class were included/excluded which makes comparison between studies difficult and might be the source of a potential misclassification bias • Only two studies [8, 13] stated confidence intervals for their prevalence which makes it the interpretation of the differences difficult Eight studies [13–15, 41, 42, 45, 46] analysed data for more than one year but only four of the studies [13, 15, 45, 46] reported trends over time • All studies except one [8] looked at patients with at least one prescription of opioids to calculate prevalence of opioid prescription which is a potential underestimation of opioid use • Only two studies [8, 43] stated their inclusion criteria regarding age groups • Only three studies [8, 43, 45] reported the sex of patients included in the study

Although insurance data have advantages for researchers such as large sample sizes that yield statistically significant results, there are several methodological flaws related to the use of such data in health research. Firstly, it is often pointed out that insurance data is primarily collected for financial purposes (instead of research purposes) which makes it somewhat unsuitable for thoroughly exploring important health-related research questions (e.g. prevalence estimates, risk factors, aetiology, and treatment outcomes). Furthermore, results of studies based on insurance data are not likely to be generalisable to a wider population (external validity) due to study samples not being representative of the population of interest [52]. With most studies included in our review (8 out of 12) analysing data from statutory health insurances (gesetzliche Krankenversicherung—GKV)—representing only 90% of the German population—certain population groups are not included in datasets and analyses, which introduces selection bias. Therefore, no generalisability of our results is given for the group of privately insured Germans, namely civil servants, freelancers and most citizens earning annual salaries above a certain threshold (€57,600 per year in 2017). Lastly, the use of insurance claims data incorporates a bias in terms of (over)estimation of the prevalence of opioid use, since it does not provide information on how much of the opioids prescribed were actually consumed by patients. Only four studies—published before 2000—reported total numbers of prescriptions within their samples and additionally, prescriptions per person [42, 44, 46, 47]. Eight papers only accounted for patients with at least one prescription, which may be a possible underestimation of the prevalence [8, 13–15, 40, 41, 43, 45].

A further limitations of this review is the observed heterogeneity in study methodology. This is reflected by big differences in sample selection between reviewed studies. Only three studies used randomisation in their sample selection process [8, 13, 45] and only two papers checked whether chosen samples were representative of the German population [8, 14]. Schubert et al. and Ihle et al. [13, 45] standardised their calculated annual treatment prevalence to the population of Germany on 31 December of 1999 and the previous year respectively. Buth et al. [15] chose the entire population of all four states included in the sample as a denominator for their prevalence calculations and extrapolated their results since only 88% of the population is covered by their data source. One study excluded two patients from the sample after primary analysis due to treatment for opioid dependence rather than for pain conditions [44]. One study did not describe their sample selection process [46].

Six studies referred to the entire German population [8, 14, 40, 41, 43, 46]. Four of them used data from only one statutory health insurance [8, 14, 40, 41]. A sampling bias could be seen in the six studies using data from specific regions within Germany, which makes it difficult to draw inferences about the German population as a whole [13, 15, 42, 44, 45, 47]. None of the studies checked for representativeness of the samples selected regarding age or sex (selection bias).

Furthermore, case ascertainment varied greatly. Five studies calculated prevalence of strong opioid use, with only Zenz et al. solely including cancer patients [40, 42, 44, 46, 47]. Marschall et al. [8] solemnly referred to CNCP patients with LTOT and Werber et al. [14] only investigated chronic pain conditions. Five studies reported the prevalence of all opioid prescriptions regardless of opioid classification or type of pain [13, 15, 41, 43, 45]. However, opioids included in these studies differed: two studies excluded codeine, methadone and levomethadone [13, 45], one included all opioids [14], and one only excluded methadone and polamidone [8]. One study additionally included the use of benzodiazepines and z-substances in the analysis [15]. Jacob et al. [43] did not investigate opioid use but researched the use of pain medication in the UK, Germany and France in general. Hoer et al. [41] primarily included all insureds with opioid prescriptions in their study but focused on the prevalence of transdermal and oral opioids.

As a result of the diversity in terms of study design and outcomes, findings from most of the reviewed studies are temporally and geographically fragmented. Therefore, they cannot be considered as representative of the German general outpatient population, nor be generalised to a particular period of time or geographical area. Nevertheless, they bring up some evidence allowing us to create a—so far missing—first summary of opioid prescribing practices in Germany.

## Conclusion

Over the last decades, pain management has been significantly improved in developed countries, notably due to a wider availability of opioid pain relievers for CNCP treatment [53]. On average, the prevalence of opioid prescriptions grew by almost 110% between 2002 and 2007 across the OECD. In Europe, changes in opioid consumption have been mainly characterised by increasing use of tramadol, fentanyl and oxycodone. The rapid growth of the opioid market, combined with described changes in prescription patterns, led to opioid use being on the agenda of public health professionals worldwide [54].

In this context, the aim of this review was to assess and compare the evidence on opioid prescription in Germany, to discuss relevant literature and to evaluate trends in prescribing and potential differences in prescription patterns.

Despite aforementioned limitations and the restricted comparability between studies, it can be stated that both, number of opioid prescriptions overall and number of people receiving opioid treatment, have increased during the last decades. Findings from our review are therefore consistent with previous research in the field, according to which Germany is the second largest consumer of opioid pain relievers in Europe behind the United Kingdom and ahead of Spain [55]. Most opioid prescriptions nowadays involve strong opioids and are given out to patients with non-cancer pain, with Fentanyl being the most prescribed strong opioid in outpatient settings. However, even though patterns of opioid prescription follow similar trends than other developed countries, there are no signs of an opioid epidemic in Germany so far, especially considering that the number of opioid-related deaths has remained stable since 2006 [50, 51]. Therefore, this review could currently not find a need for urgent health policy interventions regarding opioid prescription practices. However, critical gaps in the literature remain and more well-conducted data collection and research is needed to make more reliable judgements.

## Supporting information

S1 Table Quality assessment of included literature using the EPHPP. (DOCX)

S1 File PRISMA checklist. (DOCX)

S2 File Protocol for systematic review. (DOCX)

## Abbreviations

AOK: Allgemeine Ortskrankenkasse
AWMF: Arbeitsgemeinschaft der Wissenschaftlichen Medizinischen Fachgesellschaften e.V.
BtM: Betäubungsmittel
BtMG: Betäubungsmittelgesetz
BtMVV: Betäubungsmittelverschreibungsverordnung
CCP: Chronic cancer pain
CNCP: Chronic non-cancer pain
DALY: Disability-adjusted life year
DDD: Defined daily doses
DPM: Der Deutsche Pharmamarkt
G20: Group of 20
GDP: Gross domestic product
GDP (PPP): Gross domestic product at purchasing power parity
IMF: International Monetary Fund
LTOT: Long-term opioid treatment
NARZ: Norddeutsches Apothekenrechenzentrum
OECD: Organisation for Economic Co-operation and Development
UK: United Kingdom
UN: United Nations
US: United States
WHO: World Health Organisation
