# Supplementary material for: Opioid prescription patterns in Germany and the global opioid epidemic: Systematic review of available evidence
Source: PLoS One. 2019 Aug 28;14(8):e0221153. doi: 10.1371/journal.pone.0221153 (PMC6713321; doi:10.1371/journal.pone.0221153)
Supplement: S1 Table — (DOCX) [file pone.0221153.s001.docx]

**S1 Table.** **Quality assessment of included literature using the EPHPP.**

| Citation | Buth 2017 [15] | Hoer 2008 [41] | Hoffmann 2012 [40] | Ihle 2012 [45] | Jacob 2018 [43] | Lindena 1996 [46] | Marschall 2016 [8] | Schubert 2013 [13] | Sorge 1990 [47] | Werber 2015 [14] | Willweber-Strumpf 1992 [44] | Zenz 1995 [42] |
| --- | --- | --- | --- | --- | --- | --- | --- | --- | --- | --- | --- | --- |
| Study design | CSS^b^ | CSS^a^ | CSS^a^ | CSS^b^ | CSS^a^ | CSS^b^ | CSS^a^ | CSS^b^ | CSS^b^ | CSS^b^ | CSS^a^ | CSS^a^ |
| Sample source | Prescription data / Pharmacy Computing Centre (NARZ) (North Germany, 2005 to 2011) | Insurance claims data / Statutory health insurance (Germany, 2000 to 2003) | Insurance claims data / BARMER GEK (Germany, 2011) | Insurance claims data / AOK Hesse (Hesse, 2000 to 2009) | Patient records / Disease Analyzer database (Germany, France and UK, 2016) | Mixed sources (Germany, 1990 to 1996) | Insurance claims data / BARMER GEK (Germany, 2012) | Insurance claims data / AOK Hesse (Hesse, 2000 to 2010) | Insurance claims data / AOK Hannover (Hannover, 1985 & 1988) | Insurance claims data / BARMER GEK (Germany, 2006 to 2010) | Insurance claims data / AOK Bochum (Bochum, 1989 to 1990) | Computerised patient records data (West Germany, 1990 to 1993) |
| Number of subjects | ≈ 11,000,000 | 1 534 034 | 9 100 000 | 326,598 (2000) 264,982 (2009) | 4 270 142 | 1 218 436 | 870 000 | 326,554 (2000) 265,213 (2010) | 322,467 (1985) 325,506 (1988) | 6 800 000 | 92 842 | 1 104 435 |
| A. Selection Bias | 1 | 3 | 3 | 3 | 1 | 1 | 3 | 3 | 3 | 3 | 3 | 1 |
| B. Design | 2 | 3 | 3 | 3 | 2 | 2 | 3 | 3 | 3 | 3 | 3 | 2 |
| C. Confounders | 3 | 2 | 1 | 1 | 1 | 2 | 1 | 2 | 1 | 3 | 3 | 1 |
| D. Blinding | 1 | 1 | 1 | 1 | 1 | 1 | 1 | 1 | 1 | 1 | 1 | 1 |
| E. Data collection methods | 2 | 3 | 3 | 3 | 2 | 2 | 3 | 3 | 3 | 3 | 3 | 2 |
| F. Withdrawals & Dropouts | 1 | 1 | 1 | 1 | 1 | 1 | 1 | 1 | 1 | 1 | 1 | 1 |
| Overall score | **2** | **3** | **3** | **3** | **1** | **1** | **3** | **3** | **3** | **3** | **3** | **1** |

Quality indicators based on the EPHPP: 1 = “strong” rating of component; 2 = “moderate” rating of component; 3 = “weak” rating of component; N/A = Only one time point analysed
Overall score based on: 1 = Strong (no “weak” ratings); 2 = Moderate (one “weak” rating); 3 = Weak (two or more “weak” ratings)

^a^ Retrospective cross-sectional study

^b^ Retrospective repeated measures cross-sectional study
