# Supplementary material for: Opioid prescription patterns in Germany and the global opioid epidemic: Systematic review of available evidence
Source: PLoS One. 2019 Aug 28;14(8):e0221153. doi: 10.1371/journal.pone.0221153 (PMC6713321; doi:10.1371/journal.pone.0221153)
Supplement: S2 File — (DOCX) [file pone.0221153.s003.docx]

Version 3, March 2011

Systematic Review Protocol & Support Template*

* Procotol template retrieved from PROSPERO (International prospective register of systematic reviews; www.crd.york.ac.uk)

| **Title of the review** | Opioid prescription patterns in Germany and the global opioid epidemic: systematic review of available evidence |
| --- | --- |
| **First reviewer** | Bastian Rosner |
| **Team of reviewers** | Jessica Neicun & Justin C. Yang |
| **Supervisor/Project PI** | Andres Roman-Urrestarazu |
| **Clinical Portfolio Group** | - |
| **Project title (if different from review title)** | - |

| **Support** – please state if advice/training or personnel required at each stage | |
| --- | --- |
| **SR overview** | Bastian Rosner & Jessica Neicun |
| **Protocol development** | Bastian Rosner & Andres Roman-Urrestarazu |
| **Literature searching** | Bastian Rosner, Jessica Neicun, Justin Yang |
| **Quality appraisal** | Jessica Neicun & Andres Roman-Urrestarazu |
| **Data Extraction** | Bastian Rosner & Jessica Neicun |
| **Synthesis** | - |

| **1. Background to review**  Brief introduction to the subject of the review, including rationale for undertaking the review and overall aim |
| --- |
| Opioids are one of the most important and effective classes of drugs in pain medicine (Rosenblum et al, 2008; Cicero et al, 2017; Noble et al, 2010); with a key role in modern anaesthesia, palliative care, emergency medicine and specialised pain management (Noble et al, 2010; Gilson et al, 2011; Freye, 2010). In 2016, North America, Oceania and Western Europe reported an average consumption of over 10,000 defined daily doses (DDD) of opioid analgesics (INCB, 2017). Opioids pose a serious risk of addiction and abuse. Their long-term use still remains one of the biggest concerns about opioid treatment, since higher doses and more prolonged continuous use increase the probability of adverse effects, habituation and dependence (Marschall et al, 2016; Rivat et al, 2016; Sullivan et al, 2013).  What was seen by some as the unmet need for pain management in chronic pain patients caused by reluctance to use opioids considering their addiction potential (Rivat et al, 2016; Schubert et al, 2013; Buth et al, 2017), has become an upsurge in numbers of opioid prescriptions through pharmaceutical market access strategies and policy-making, now affecting G20 countries severely and especially so in chronic non-cancer pain (CNCP) patients (Vashishtha et al, 2017; Hauser et al, 2016; Baker, 2017; Van Zee; 2009). The increase of opioid prescription over the past decade has led to higher numbers of prescription opioid misuse, abuse and opioid-related death cases in most developed OECD countries around the world (Hauser et al, 2016; OECD, 2018). For example, the Global Burden of Disease Study showed a 22.3% increase in global opioid use disorder-related DALYs (disability-adjusted life years) between 2005 and 2015. Drug use disorders ranked as the 8^th^ most common cause of premature death in the US in 2016 (IHME, 2015). The Canadian government reported an 81% increase of accidental deaths involving fentanyl or fentanyl analogues between 2016 and 2017 (Government of Canada, 2018). Hence, there has been a shift from under-treatment of pain observed in the second half of the 20^th^ century to an opioid crisis linked to over-prescription in pain management strategies (Jones et al, 2018). In the US, roughly 21 to 29 per cent of patients with opioid prescriptions for chronic pain misuse them and an estimated 4 to 6 per cent of misusers eventually transition to heroin use (Cicero et al, 2014; Vowles et al, 2015; Carlson et al, 2016). In 2013, it was reported that about 2.4% of Australians aged 14 or older had used opioids for non-medical reasons over their lifetime (AIHW, 2016).  Whilst reliable data on the prevalence of opioid treatment is accessible for many countries, data on Germany specifically is lacking. This is despite Germany having the largest national economy in Europe, the fourth-largest by nominal gross domestic product (GDP) in the world, and fifth-largest by GDP at Purchasing Power Parity. In 2017, the country accounted for 28% of the euro area economy according to the IMF and with 82.0 million people, it has the largest population in the European Union (IHME, 2015; CIA, 2018). Consequently, and following the US and other developed economies, the opioid epidemic has recently become a point of contention with serious questioning as whether Germany was following trends of other developed countries towards an opioid crisis (Kaiser, 2018; Pharmazeutische Zeitung, 2018). According to the UN’s 2017 report on narcotic drug use, Germany has the second highest opioid consumption of the 20 most populous countries in the world (28,842 DDD/1 million people/day; based on sales data) (INCB, 2017).  It is crucial to understand the current prevalence of opioid prescription in Germany in order to avoid a further rise of opioid misuse and opioid-related disorders. So far, the only effective approach to this national (and international) public health problem has been to design primary population level data collection strategies to analyse current and past trends to develop sufficient long-term prevention strategies.  Aim  Considering the lack of evidence-based strategies from long-term studies on the German population in relation to the administration of opioids (Werber et al, 2015); the aim of this paper is to assess and discuss any relevant published literature. The focus of this work is on the descriptive epidemiology of opioid prescriptions in outpatients in Germany. In order to question the current epidemiology of prescription opioids in Germany, a systematic literature review will be conducted to evaluate, compare and appraise relevant studies, to assess trends in prescribing and potential differences in prescription patterns. Gathered information will be used to make judgements regarding the potential need for public health and health policy action. |

| **2. Specific objectives** |
| --- |
| 1. To identify and appraise the evidence base regarding opioid prescriptions in outpatients in Germany. 2. To identify time trends, regional differences, differences regarding certain classes of opioids, or underlying diagnoses for opioid prescriptions. 3. To summarise information to make recommendation for public health and health policy professionals regarding the current need for further restriction of opioid prescription in Germany. |

| **3. a) Criteria for including studies in the review**  If the PICOS format does not fit the research question of interest, please split up the question into separate concepts and put one under each heading | |
| --- | --- |
| 1. **Population, or participants and conditions of interest** | Outpatients in Germany with special narcotic prescription opioids; any age, any gender, any type of prescription opioid, any diagnosis |
| 1. **Interventions or exposures** | People who received at least one opioid requiring a special narcotic prescription |
| 1. **Comparisons or control groups** | - |
| 1. **Outcomes of interest** | Prevalence of opioid prescription |
| 1. **Setting** | Outpatient setting, Germany |
| 1. **Study designs** | Any study design for primary data analysis: would expect to be observational or cohort studies rather than RCT’s |

| **3. b) Criteria for excluding studies not covered in inclusion criteria** Any specific populations excluded, date range, language, whether abstracts or full text available, etc. |
| --- |
| - Full-text not accessible at University of Cambridge - Languages other than English or German - Studies conducted in other German-speaking countries - Restriction to children or adolescents - Studies strictly referring to one specific opioid (e.g. tramadol, fentanyl) - Studies solely referring to hospital opioid use - Studies (reviews) exclusively reporting results of papers already included in essay |

| **4. Search methods** | |
| --- | --- |
| **Electronic databases**  Please list all databases that are to be searched and include the interface (eg NHS, EBSCO, etc) and date ranges searched for each | - PubMed - University of Cambridge Web of Science |
| **Other methods used for identifying relevant research**  ie contacting experts and reference checking | Reference checking and hand searching of these. Manually searching Google.com for grey literature |
| **Journals hand searched**  If any are to be hand searched, please list which journals and date searched from, including a rationale. | - |

| **5. Methods of review** | |
| --- | --- |
| **Details of methods**  Number of reviewers, how agreements to be reached and disagreements dealt with, etc. | Two levels of screening by three independent researchers are used on all citations. Bastian Rosner as first reviewer, Jessica Neicun as second and Justin Christopher Yang as third reviewer. If there will be any doubt, the article will be retained for the next level of scrutiny. Disagreements are resolved by face-to-face discussion, leading to a consensus judgement. |
| **Quality assessment**  Tools or checklists used with references or URLs | The Effective Public Health Practice Project Tool (EPHPP) will be used to assess and compare the quality of included studies. |
| **Data extraction**  What information is to be collected on each included study. If databases or forms on Word or Excel are used and how this is recorded and by how many reviewers | The data extracted from each study comprised lead author, journal name, sample characteristics, study region, study design, follow-up details, primary data source and information on opioid prescription prevalence as well as on confounders. Data extraction form in Word document; Endnote to be used to keep track of references; Reviewer number 1 (BR) will review first, followed by reviewer number 2 (JN) and reviewer number 3 (JCY), which will be done independently. Disagreements will be resolved by face-to-face discussion. |
| **Narrative synthesis**  Details of what and how synthesis will be done | Narrative synthesis of the data will be done and will be carried out, addressing the following elements:   1. Results on case ascertainment and general reporting of each study 2. Specific results on prevalence, focusing on prevalence by age, sex, time trends, region, diagnosis 3. Summary of results 4. Assessing the robustness of the synthesis and the evidence base |
| **Meta-analysis**  Details of what and how analysis and testing will be done. If no meta-analysis is to be conducted, please give reason. | A Meta-analysis is not planned since a lot of heterogeneity between studies is expected and would mitigate the appropriateness of a meta-analysis. |
| **Grading evidence**  System used, if any, such as GRADE | N/A |

| **6. Presentation of results** | |
| --- | --- |
| **Additional material**  Summary tables, flowcharts, etc, to be included in the final paper | - Protocol - Prisma Checklist - Flow chart of literature search - Table with eligibility criteria - Summary tables for results - Graphs of subgroup analyses included in the final review - Table of limitations of review |
| **Outputs from review**  Papers and target journals, conference presentations, reports, etc | - Term paper as part of requirement for the degree of MPhil Public Health (University of Cambridge) - Report and presentation to MPhil peers - Paper in high quality medical journal |

| **7. Timeline for review – when do you aim to complete each stage of the review** | |
| --- | --- |
| **Protocol** | 2 weeks |
| **Literature searching** | 3 weeks |
| **Quality appraisal** | 1 week |
| **Data extraction** | 1 week |
| **Synthesis** | 3 weeks |
| **Writing up** | 4 weeks |
